# rMCP-2, the Major Rat Mucosal Mast Cell Protease, an Analysis of Its Extended Cleavage Specificity and Its Potential Role in Regulating Intestinal Permeability by the Cleavage of Cell Adhesion and Junction Proteins

**DOI:** 10.1371/journal.pone.0131720

**Published:** 2015-06-26

**Authors:** Zhirong Fu, Michael Thorpe, Lars Hellman

**Affiliations:** 1 Education Ministry Key Laboratory for Biomedical Engineering, Zhejiang University, 38 Zheda Road, Hangzhou, Zhejiang, China; 2 Department of Cell and Molecular Biology, Uppsala University, Uppsala, The Biomedical Center, Box 596, SE-751 24 Uppsala, Sweden; INRA, FRANCE

## Abstract

Mast cells of the rat intestinal mucosa express three chymotryptic enzymes named rMCP-2, -3 and 4. rMCP-2, the most abundant of these enzymes, has been shown to increase the permeability of the intestinal epithelium, most likely by cleavage of cell adhesion and junction proteins and thereby play a role in intestinal parasite clearance. However, no target for this effect has yet been identified. To address this question we here present its extended cleavage specificity. Phage display analysis showed that it is a chymase with a specificity similar to the corresponding enzyme in mice, mMCP-1, with a preference for Phe or Tyr in the P1 position, and a general preference for aliphatic amino acids both upstream and downstream of the cleavage site. The consensus sequence obtained from the phage display analysis was used to screen the rat proteome for potential targets. A few of the most interesting candidate substrates were cell adhesion and cell junction molecules. To see if these proteins were also susceptible to cleavage in their native conformation we cleaved 5 different recombinant cell adhesion and cell junction proteins. Three potential targets were identified: the loop 1 of occludin, protocadherin alpha 4 and cadherin 17, which indicated that these proteins were at least partly responsible for the previously observed prominent role of rMCP-2 in mucosal permeability and in parasite clearance.

## Introduction

Mast cells (MCs) are resident tissue cells of hematopoietic origin that are distributed along both external and internal surfaces of the body. They are frequently found in connective tissue of the skin and around blood vessels and nerves as well as in the mucosa of the airways and intestine. Two major subpopulations of MCs have been identified and have been named connective tissue (CTMC) and mucosal MCs (MMCs), based on their tissue location [1]. Mucosal MCs are more T-cell dependent and increase in numbers relatively rapidly after parasite infection in response to TGF-β and IL-9 [2–4]. Both types of MCs are able to rapidly exocytose their cytoplasmic granules following activation, which results in the release of a number of pre-stored inflammatory mediators [5]. The majority of proteins found in these granules are serine proteases, which can generally be subdivided into chymases and tryptases [6–8]. Chymases are chymotrypsin-like and cleave substrates after aromatic amino acids (aa), whereas tryptases are trypsin-like enzymes with preference for positively charged aa at their cleavage site [6–8]. Mucosal MCs in rats and mice only express chymases and no tryptic enzymes [9, 10]. This is in contrast to human MMCs, which primarily express tryptases. Phylogenetic analyses of the chymases have led to the identification of two distinct subfamilies: the α-chymases and the β-chymases [9, 11]. The α-chymases are found as a single gene in all species investigated, except for ruminants. In cattle and sheep two very similar α-chymase genes have been identified [12]. The β-chymases have only been identified in rodents with one potential exception, the CMA2 gene in dogs, which shows some similarities to the β-chymases [13]. All three rat MMC proteases, rMCP-2, -3 and -4, belong to the β-chymase subfamily [9].

In mice and rats MMCs have been shown to increase in numbers quite dramatically after infection by intestinal parasites, and when the infection is cleared, the MMC numbers return to normal after a few weeks [10, 14]. This indicates a role of MMCs in parasite clearance and puts focus on what factors produced by MMCs are important for this potential role in parasite defense. One finding that indicates a prominent role of MMC chymases is when injected intravenously, rMCP-2 induces increased epithelial permeability in the intestinal region and a translocation of Evans blue labelled human serum albumin from the blood vessels into the intestinal lumen within minutes [15]. Triggering of MC release by parasite antigen in animals previously exposed to the parasite also leads to massive release of rMCP-2, its appearance in the intestinal lumen and increased permeability within minutes after challenge. The increased intestinal permeability in turn leads to efflux of components of the immune system such as complement components, immunoglobulins and also inflammatory cells including eosinophils, neutrophils and macrophages. These soluble components and cells are thought to increase the capacity to combat infections by intestinal parasites. Of particular interest here are helminth (worm) parasites, which are large and thereby relatively difficult for the immune system to handle. In line with the suggested role of these proteases in the defense against intestinal parasites a mMCP-1 knock out results in an increased time of clearance of certain helminthes [16]. One of the major questions in the field has been the target for these enzymes and how the cleavage of a few selected cell surface molecules can lead to this increased permeability. In order to address this question we have determined the extended cleavage specificity of the major MMC protease in the rat rMCP-2, which is the protease thought to be the primary player in this phenomenon [14]. A detailed analysis of the extended specificity can help in defining potential *in vivo* substrates by screening of the entire rat genome for potential targets with a well-defined sequence. This can reduce the number of potential targets and focus the study on the most likely of these molecules. Among the most interesting targets identified in this screening were a few epithelial and endothelial cell junction proteins. Recombinant protein for five such proteins were analyzed for their sensitivity to cleavage by rMCP-2, which resulted in efficient cleavage of three of them, indicating that may be some of the prime targets for this enzyme.

## Materials and Methods

### Production, purification and activation of recombinant rMCP-2

The rMCP-2 sequence was designed and ordered as designer gene from GenScript (Piscataway, NJ, USA). The rMCP-2 sequence was subsequently transferred to a pCEP-Pu2 vector, used for expression in mammalian cells [17]. The enzyme was produced as an inactive recombinant protein, with an N-terminal His_6_-tag followed by an EK site. HEK 293 EBNA cells were grown to 70% confluency in a 25 cm^3^ tissue culture flask (BD VWR) with Dulbecco’s Modified Eagles Medium (DMEM) (GlutaMAX, Invitrogen, Carlsbad, CA, USA) supplemented with 5% fetal bovine serum (FBS) and 50 μg/ml gentamicin. Following DNA (25 μg of rMCP-2 in pCEP-Pu2) transfection with lipofectamine (Invitrogen, Carlsbad, CA, USA), puromycin was added to the DMEM (0.5 μg/ml) to select for cells which had taken up the DNA. Heparin was also added to the medium (5 μg/ml) to enhance yield, as these positively charged proteases tend to bind cell surfaces and plastic and heparin can then reduce the loss of the recombinant protein. Cells were expanded and conditioned media collected.

To purify the recombinant enzyme, 750 ml conditioned media was filtered (Munktell 00H 150 mm, Falun, Sweden) and 500 μl nickel nitrilotriacetic acid (Ni-NTA) beads were added. The media with Ni-NTA beads were rotated for 45 minutes at 4°C. Subsequently, the Ni-NTA beads were collected by centrifugation and transferred to a column containing a glass filter (Sartorius, Goettingen, Germany). After washing with PBS tween 0.05% + 10 mM imidazole + 1 M NaCl, the recombinant protein was eluted in PBS tween 0.05% + 100 mM imidazole fractions. The first fraction volume was half the Ni-NTA bead width (200 μl) and further fractions eluted with a full bead width (400 μl). Individual fractions were run on SDS-PAGE gel, their concentrations estimated from a bovine serum albumin standard (BSA) and the most concentrated were pooled and kept at 4°C.

The recombinant rMCP-2 initial concentration was determined by SDS-PAGE and the level of EK (Roche, Mannheim, Germany) adjusted for activation of the enzyme. A relative concentration was activated depending when it was needed, where for example, 70 μl of the eluted recombinant enzyme was digested with 1 μl EK for 3 hrs at 37°C. The activated fractions were stored at 4°C until use.

### Determination of cleavage specificity by phage-displayed nonapeptide library

A library of 5x10^7^ unique phage-displayed nonameric peptides was used to determine the cleavage specificity of rMCP-2 as previously described [18–20]. In these T7 phages, the C-terminus of the capsid protein 10 were manipulated to contain a nine aa long random peptide followed by a His_6_-tag [18]. An aliquot of the amplified phages (~10^9^ pfu) were bound to 100 μl Ni-NTA beads by their His_6_-tags for 1 h at 4°C under gentle agitation. Unbound phages were removed by washing ten times in 1.5 ml 1 M NaCl, 0.1% Tween-20 in PBS, pH 7.2, and two subsequent washes with 1.5 ml PBS. The beads were finally resuspended in 1 ml PBS. Activated arMCP-2 (~0.6 μg) was added to the resuspended beads and left to digest susceptible phage nonapeptides under gentle agitation at room temperature over night. PBS without protease was used as control. Phages with a random peptide that was susceptible to protease cleavage were released from the Ni-NTA matrix, and the supernatant containing these phages was recovered. To ensure that all of the released phages were recovered the beads were resuspended in 100 μl PBS (pH 7.2) and the supernatant, after mixing and centrifugation, was added to the first supernatant. To ensure that the His_6_-tags had been hydrolyzed on all phages recovered after protease digestion, 15 μl fresh Ni-NTA agarose beads were added to the combined phage supernatant and the mixture agitated for 15 min followed by centrifugation. A control elution of the phages still bound to the beads, using 100 μl 100 mM imidazole showed that at least 1 x 10^8^ phages were attached to the matrix during each selection. Ten μl of the supernatant containing the released phages was used to determine the amount of phages detached in each round of selection. Dilutions of the supernatant were plated in 2.5 ml 0.6% top agarose containing 300 μl of *E*. *coli* (BLT5615), 100 μl diluted supernatant and 100 μl 100mM IPTG. The remaining volume of the supernatant was added to a 10 ml culture of BLT5615 (OD ~0.6). The bacteria had 30 min prior to phage addition been induced to produce the T7 phage capsid protein by the addition of 100 μl 100 mM IPTG to the culture. The bacteria lysed approximately 75 minutes after phage addition. The lysate was centrifuged to remove cell debris and 500 μl of the phage sub-library was added to 100 μl fresh Ni-NTA beads, to start the next round of selection. After binding the sub-library for 1 h at 4°C under gentle agitation, the Ni-NTA beads were washed 15 times in 1.5 ml 1 M NaCl, 0.1% Tween-20 in PBS, pH 7.2, followed by two subsequent washes with 1.5 ml PBS.

Following five rounds of selection, 120 plaques were isolated from LB plates after plating in top agarose. Each phage plaque, corresponding to a phage clone, was dissolved in phage extraction buffer (100 mM NaCl and 6 mM MgSO_4_ in 20 mM Tris-HCl pH 8.0) and vigorously shaken for 30 minutes in order to extract the phages from the agarose. The phage DNA was then amplified by PCR, using primers flanking the variable region of the gene encoding the modified T7 phage capsid-protein. After amplification, PCR fragments of 96 clones were sent to GATC Biotech in Germany for sequencing.

### Generation of a consensus sequence from sequenced phage inserts

Phage insert sequences were aligned by hand assuming a preference for aromatic aa in position P1. Sequences with only one aromatic aa were aligned first and sequences with more than one possible cleavage site were then aligned to fit this pattern. Amino acids with similar characteristics were grouped together as follows: aromatic aa (Phe, Tyr, Trp); negatively charged aa (Asp, Glu); positively charged aa (Lys, Arg), small aliphatic aa (Gly, Ala); larger aliphatic aa (Val, Leu, Ile, Pro), hydrophilic aa (Ser, Thr, His, Asn, Gln, Cys, Met). The nomenclature by Schechter and Berger [21] was adopted to designate the aa in the substrate cleavage region, where P1-P1' corresponds to the scissile bond.

### Generation of recombinant substrates for the analysis of the cleavage specificity

A new type of substrate was developed to verify the results obtained from the phage display analysis. Two copies of the *E*. *coli* thioredoxin gene were inserted in tandem into the pET21 vector for bacterial expression. In the C-terminal end a His_6_- tag was inserted for purification on Ni^2+^ IMAC columns. In the linker region, between the two thioredoxin molecules, the different substrate sequences were inserted by ligating double stranded oligonucleotides into two unique restriction sites, one BamHI and one SalI site. The sequences of the individual clones were verified after cloning by sequencing of both DNA strains. The plasmids were then transformed into the *E*.*coli* Rosetta gami strain for protein expression (Novagen, Merck, Darmstadt, Germany). A 10 ml overnight culture of the bacteria harbouring the plasmid was diluted 10 times in LB + Amp and grown at 37°C for 1–2 hours until the OD (600 nm) reached 0.5. IPTG was then added to a final concentration of 1 mM. The culture was then grown at 37°C for an additional 3 h under vigorous shaking, after which the bacteria were pelleted by centrifugation at 3500 rpm for 12 minutes. The pellet was washed once with 25 ml PBS + 0.05% Tween 20. The pellet was then dissolved in 2 ml PBS and sonicated 6 x 30 seconds to open the cells. The lysate was centrifuged at 13000 rpm for 10 minutes and the supernatant was transferred to a new tube. Five hundred μl of Ni-NTA slurry (50:50) (Qiagen, Hilden, Germany) was added and the sample was slowly rotated for 45 min at 4°C. The sample was then transferred to a 2 ml column and the supernatant was allowed to slowly pass through the filter leaving the Ni-NTA beads with the bound protein in the column. The column was then washed four times with 1 ml of washing buffer (PBS + 0.05% Tween + 10 mM Imidazole + 1 M NaCl). Elution of the protein was performed by adding 150 μl elution buffer followed by five 300 μl fractions of elution buffer (PBS + 0.05% Tween 20 + 100 mM Imidazole). Each fraction was collected individually. Ten μl from each of the eluted fractions was then mixed with 1 volume of 2 x sample buffer and 1 μl β-mercapto-ethanol and then heated for 3 min at 80°C. The samples were analyzed on a SDS bis tris 4–12% PAGE gel and the second and third fractions that contained the most protein were pooled. The protein concentration of the combined fractions was determined by Bio-Rad DC Protein assay (Bio-Rad Laboratories Hercules, CA USA). Approximately 25 μg of recombinant protein was added to each 50 μl cleavage reaction (in PBS). Ten μl from this tube was removed before adding the enzyme, the 0 minute time point. The active enzyme was then added (5 ng or 200ng (40x) of rMCP-2) and the reaction was kept at room temperature during the entire experiment. Ten μl samples were removed at the indicated time points (15 min, 45 min and 150 min) and stopped by addition of one volume of 2 x sample buffer. One μl β-mercapto-ethanol was then added to each sample followed by heating for 3 min at 80°C. Ten μl from each of these samples was then analyzed on 4–12% pre-cast SDS-PAGE gels (Invitrogen, Carlsbad, CA, USA). The gels were stained over night in colloidal Coomassie staining solution and de-stained for several hours according to previously described procedures [22].

### Separation of rat intestinal epithelium from mesenchyme

In order to reduce the complexity of the analysis and to reduce the amount of abundant proteins in rat intestinal mesenchyme, intestinal epithelium was separated from its mesenchymal crypt beds using the protocol described by Nik and Carlsson [23]. Briefly, the intestine was taken from a rat, rinsed in cold PBS and then cut into pieces of 4–5 cm in length. A dented plastic rod was inserted into a piece of intestine, secured with suture and pulled back to invert the intestine, which was then thoroughly washed with PBS. Subsequently, one end of the inverted intestine was ligated with suture and the other end fitted to the tip of a 2.5 ml plunger. The intestine was then submerged in ice-cold BD Cell Detachment Solution (BD Biosciences, Franklin Lakes, NJ USA), inflated air with plunge and kept on ice for 20 min with occasional deflation/reflation. During the process, the BD Cell Detachment Solution dissolved the basement membrane, at the meanwhile the air pressure inside the inverted intestine pushed the epithelial crypts out of the mesenchyme. Therefore, the intestinal epithelium could be collected after 20 min.

### Ethics statement

All animal experiments were approved by the Uppsala Djuretiska Nämnd (the local ethics committee) permit no C11/14. All experiments have been conduction in accordance to national and international guidelines. The only animal experiments performed in this study was to kill two rats by standard CO_2_ euthanizing and remove their intestines for analysis of the protein content of the epithelial layer by one and two dimensional gels.

### Protein sample preparation

The pieces of epithelial layer were resuspended in PBS and then evenly divided into eight Eppendorf tubes. In three tubes with epithelium active rMCP2 enzyme was added (5ul of 0.304 ug/ul), and in three of them inactive rMCP2 enzyme (5ul of 0.304 ug/ul). In two additional tubes 2-Trx recombinant substrate of rMCP2 consensus was added (95ul of 2.78 mg/ml) as internal control. In one of these tubes we added active rMCP2 and in the other we added inactive rMCP2. All the reactions were incubated for 2 h at 37°C. The intestinal tissue was then pellet by centrifugation for 5 min at 2000 r, 4°C. Lysis buffer 500 ul (8 M Urea, 2% CHAPS, 65 mM DTT, 0.5% (v/v) IPG buffer, 1mM PMSF) was then added and the sample was sonicated for 5–10 rounds of 15 seconds at ~50% power in a MSE Soniprep 150 (MSE Ltd, London UK) [24, 25]. The lysates were incubated on the shaker for 10–15 min at room temperature to solubilize proteins efficiently, and centrifuged for 20 min at 16,000 x g, 4°C to remove the insoluble components. The supernatant was collected and its protein concentration was quantified by Bio-Rad DC Protein assay (Bio-Rad Laboratories Hercules, CA USA). The protein extracting solution was aliquoted and stored at -70°C or directly loaded for SDS-PAGE and isoelectric focusing.

### SDS-PAGE analysis

To compare the proteins in rat intestinal epithelium after incubation with active or inactive rMCP2, 25 ug protein extracts prepared as above were analyzed on 4–12% pre-cast SDS-PAGE gels (Invitrogen) by Xcell SureLock Mini-Cell (Invitrogen, Carlsbad, CA, USA) with 120 V for 75min. After electrophoresis, the gels were stained overnight in colloidal Coomassie staining solution and destained with 25% (v/v) methanol in ddH_2_O for 4 fours.

### 2-D analysis

2-D analysis of rat intestinal epithelium incubated with inactive or active rMCP2 was performed with ZOOM IPGRunner Mini-cell system according to the Invitrogen standard protocol (Invitrogen, Carlsbad, CA, USA). Specifically, 7cm isoelectric focusing (IEF) IPG (pH 3–10 NL) strips (Invitrogen, Carlsbad, CA, USA) were rehydrated for 1 hour with 140 ul of IEF buffer containing an estimated 50 ug protein extract. Rehydrated strips were then focused on Zoom IPGRunn mini-cell (Invitrogen, Carlsbad, CA, USA) with the following isoelectric focusing program: 50–175 V ramp for 1h, 175 V for 15 min, 175–2000 V for 45 min, 2000 V for 30 min. After IEF, strips were equilibrated for 15 min with 10 ml 1X LDS sample buffer (Invitrogen, Carlsbad, CA, USA) containing 1ml of 10 X sample reducing agent (Invitrogen, Carlsbad, CA, USA) and subsequently alkylated for additional 15 min with 10 ml 1X LDS sample buffer containing 125 mM iodoacetamide (Sigma Aldrich, Saint Louis, MO, USA). Equilibrated strips were transferred to 4–12% pre-cast SDS-PAGE gels for the second dimensional separation by using Xcell SureLock Mini-Cell. Following electrophoresis, 2-D gels were stained with SilverQuest Staining kit (Invitrogen, Carlsbad, CA, USA). All the 2-D gels were repeated at least 3 times for each sample. Stained gels were scanned, and three well-resolved gels of each sample were selected as representative gels. Automated image analysis was then performed to detect and match protein spots by using Image Master 5.0 software (GE Healthcare-Amersham Bioscience) followed by manual editing.

### Cleavage analysis of recombinant cell adhesion and cell junction proteins

To study the sensitivity to cleavage of a panel of cell adhesion and cell junction proteins we screened the internet for potential companies that could offer such potential target molecules that had been produced in mammalian cells and thereby could have a proper folding. We identified three molecules of interest, His tagged rat intestinal cadherin 17 produced in human cells (Cdh1-7-7477R, 50 ug) from Creative BioMart (Shirley NY, USA), recombinant human protocadherin alpha 4 carrier free (6509-CA-050, 50 ug) produced in CHO cells (R&D systems Abingdon UK), and rat E-cadherin Fc chimera carrier free produced in the mouse myeloma cell line NS0 (8144-EC-050, 50 ug) (R&D systems Abingdon UK).

Several other cell adhesion and junction proteins were of interest among them occludin and claudin 7 as both of these have been shown to be expressed at high levels in mouse and/or rat intestine. However both of them are complicated membrane proteins that span the membrane 4 times and therefore are difficult to produce in a soluble form and properly folded in any expression system. We therefore decided to produce the extracellular loops that can be targets for an enzyme that act from outside of the cell. The extracellular region including a few amino acids from the outer part of the membrane spanning region was inserted in the 2x Trx vector. In order to stabilize the construct and obtain a folding similar to the bended loop structure of the native protein we inserted two cysteine residues, one in each end of the loop. These inserts were produced as designer gens by GenScript (Piscataway, NJ, USA) and then cloned into the 2x trx vector. These recombinant proteins, approximately 2.5 ug per lane, were cleaved with active recombinant rMCP-2 for up to 150 minutes and then loaded onto an SDS-PAGE gel that was stained with colloidal Coomassie blue as previously described under the cleavage of 2xTrx recombinant substrates.

## Results

### Production, purification and activation of recombinant rMCP-2

**Fig 1:**
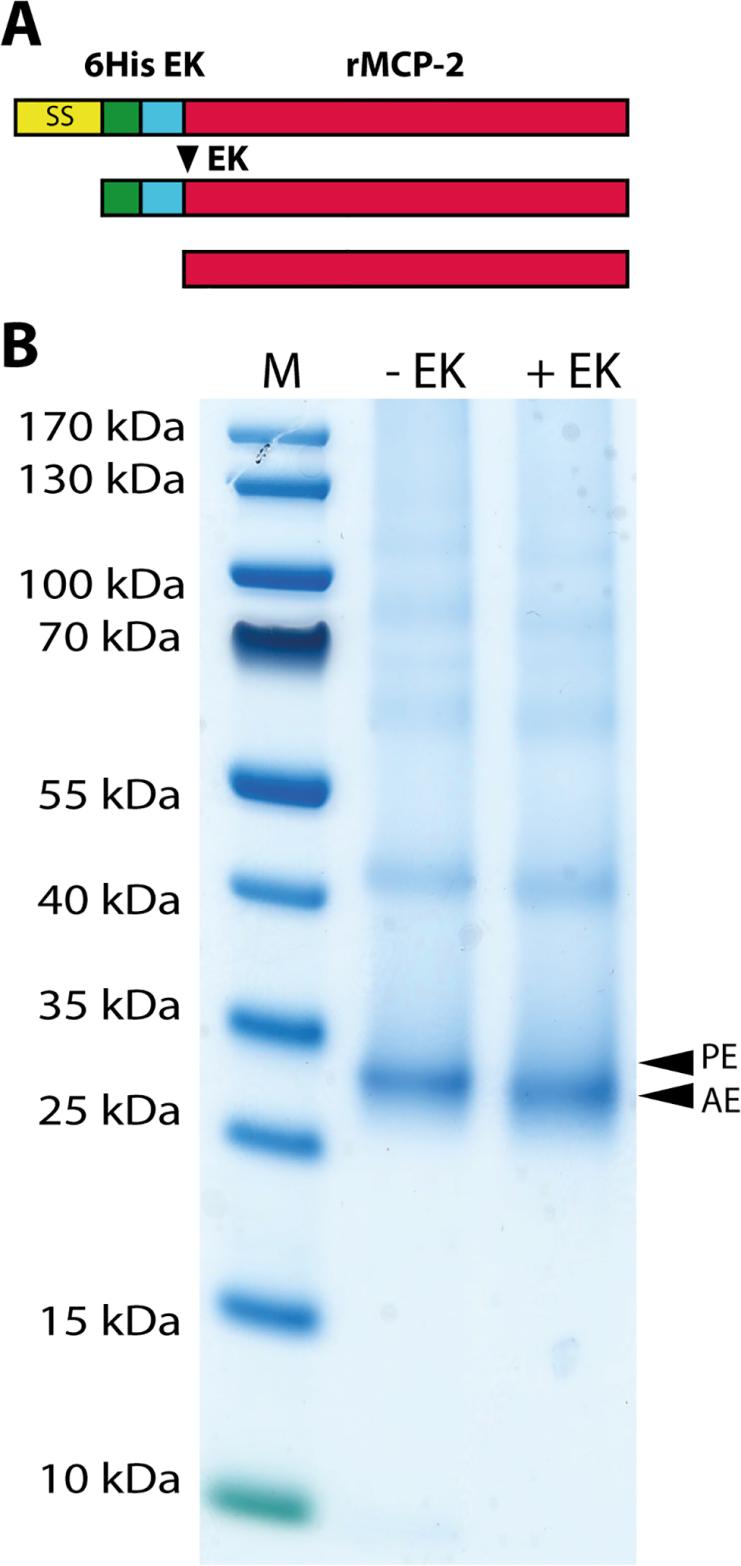
Purification and activation of recombinant rMCP-2. rMCP-2 was expressed in the human HEK 293 EBNA cell line. The proenzyme was first purified, from the conditioned media of transfected cells, on Ni-NTA beads (-EK) and then activated by removal of the His_**6**_-tag by enterokinase digestion (+EK). The purified enzyme, before and after enterokinase cleavage was analyzed on SDS-PAGE and visualized with Coomassie Brilliant Blue staining. Panel A shows schematic drawings of the initial translated product, the product after signal sequence removal and finally the active enzyme after enterokinase cleavage. Panel B shows the Coomassie blue stained SDS-PAGE gel with a size marker, the purified enzyme before and after enterokinase cleavage (-EK and +EK). The two arrows show the proenzyme (PE) and the active enzyme (AE).

A DNA construct containing the coding region for the active rMCP-2 was designed and ordered from Genscript (Piscataway, NJ, USA). This DNA fragment also contained the coding region for a His_6_-tag followed by an EK site, which was positioned N-terminally of the active rMCP-2 coding sequence. This construct was cloned into the mammalian expression vector pCEP-Pu2 for expression in HEK 293 EBNA cells [17]. The His_6_-tag facilitates purification on Ni^2+^ chelating immobilized metal ion affinity chromatography columns and cleavage with EK activates the enzyme, whilst simultaneously removing the His_6_-tag. Samples of inactive and activated protease were separated on SDS-PAGE gels in order to ensure successful removal of the His_6_-tag and the EK susceptible cleavage site (Fig 1). The inactive protease migrated as 27–28 kDa bands and the EK digested enzyme was only slightly smaller (Fig 1).

The activity of the enzyme was analyzed by cleavage of the chymotrypsin sensitive chromogenic substrate S-2586 (MeO-Suc-Arg-Ala-Tyr-pNA, Chromogenix Mölndal Sweden). The enzyme was found to have a very high activity (data not shown).

### Determination of the extended cleavage specificity by substrate phage display

Phage display technology was used to determine the extended cleavage specificity of rMCP-2. The extended specificity is the involvement of the region surrounding the actual cleavage site, usually involving 3–4 amino acids both upstream and downstream of the cleavage site. The library used for the phage display analysis is based on the *E*.*coli* T7 phage and contains approximately 50 million individual phage clones. Each phage clone expresses a unique sequence of 9 random amino acids, followed by a His_6_-tag in the C-terminus of capsid protein 10. Thereby the phages display a random nonamer on their surface and by interactions of the His_6_-tag the phages can be immobilized on Ni-NTA agarose beads. The active rMCP-2 was used to screen the phage library for peptides susceptible to cleavage. After the first selection step (biopanning), the phages, released by digestion of nona-peptides, were amplified in *E*. *coli* and subjected to additional biopannings. Selections of nonamers, susceptible to cleavage by rMCP-2, were performed over 5 biopannings, after which it induced the release of 74 000 times more phages compared to a PBS control. The very high ratio, the best that we have obtained, is probably related to the very high specific activity of the enzyme.

**Fig 2:**
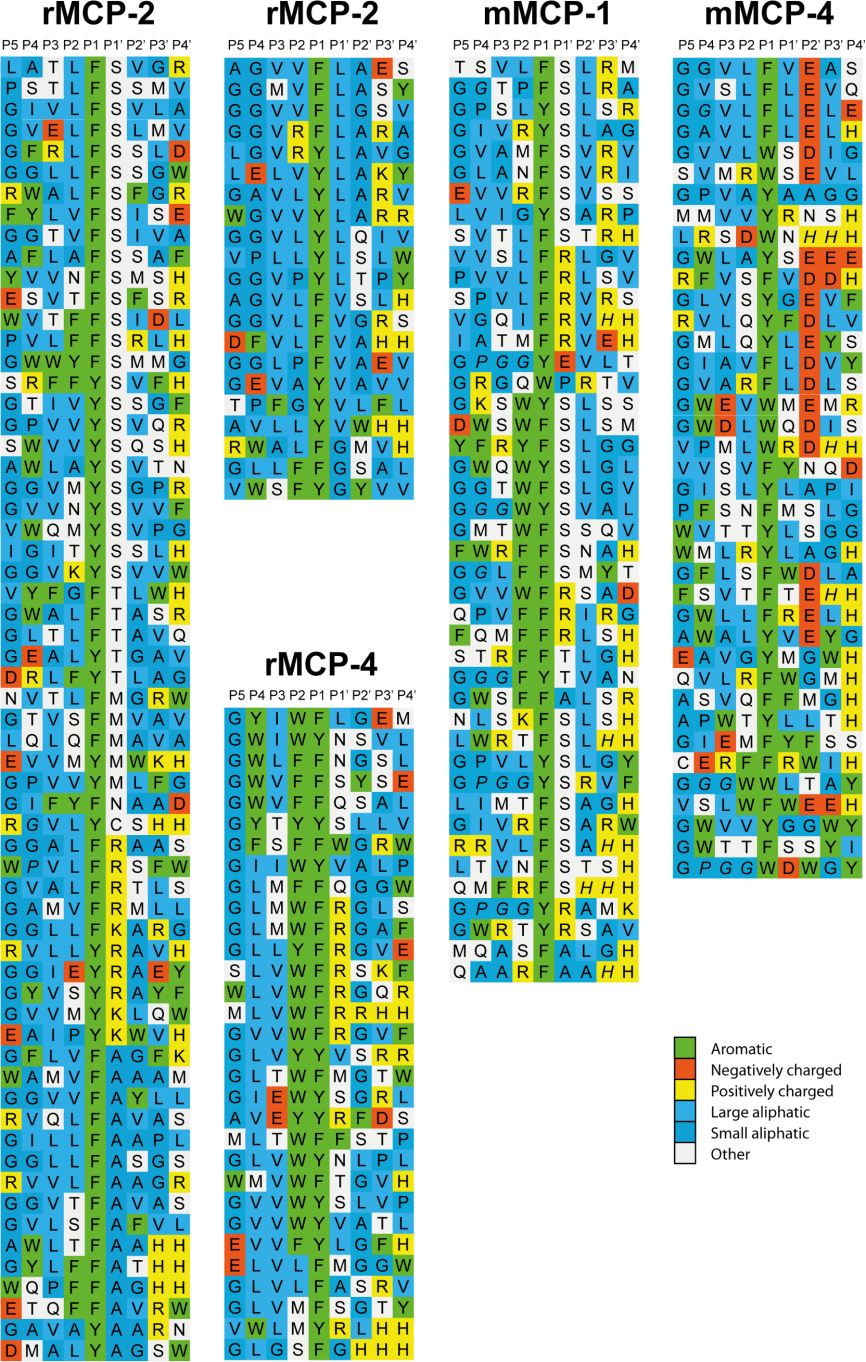
Phage displayed nonamers susceptible to cleavage by rMCP-2 after five biopannings. After the last selection step (round 5), phages released by proteolytic cleavage of the protease were isolated and the sequences encoding the nonamers were determined. The general sequence of the T7 phage capsid proteins are PGG(X)_**9**_HHHHHH, where (X)_**9**_ indicates the randomized nonamers. The protein sequences were aligned into a P4-P4´ consensus, where cleavage occurs between positions P1 and P1´. The aa are color coded according to the side chain properties as indicated in the figure, bottom right corner. For comparison the previously determined phage display results from rMCP-4, mMCP-1 and mMCP-4 are included in the figure [**18**, **20**, **34**].

**Fig 3:**
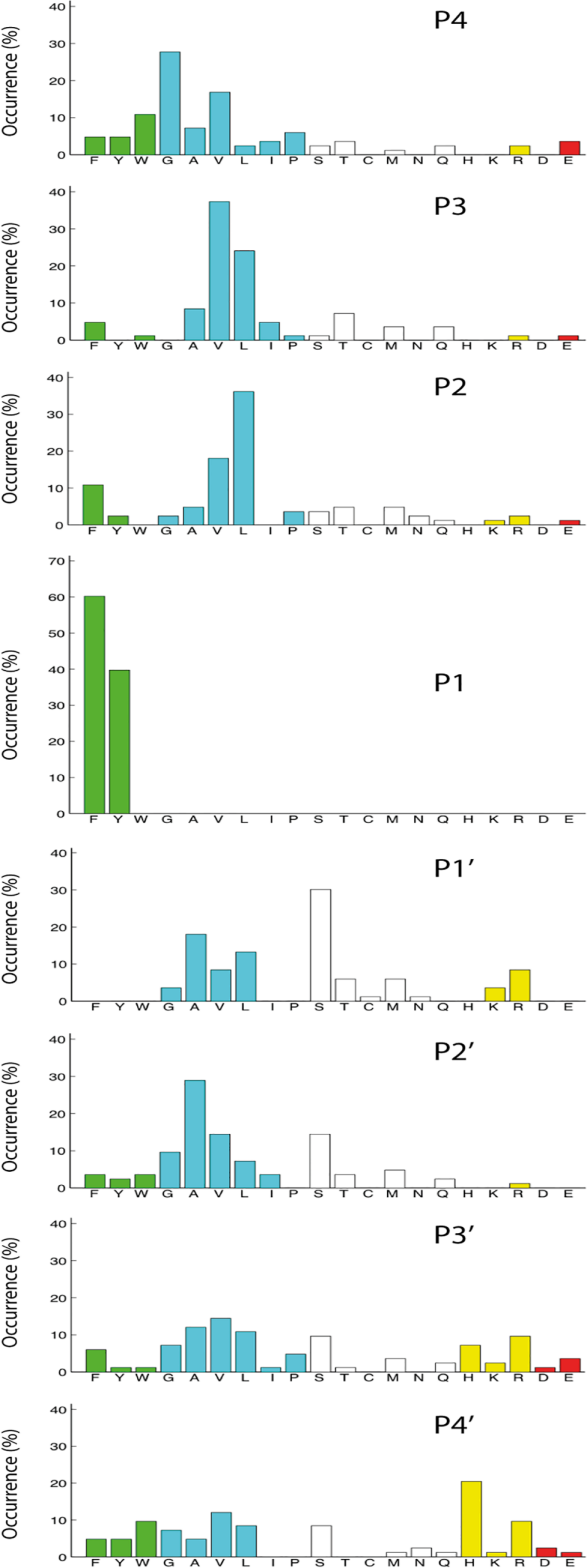
Distribution of aa in positions P4 to P4´ in phage displayed nonamers cleaved by rMCP-2 after five biopannings. Based on the alignment in Fig 2 the percentage of each amino acid present in each position P4 to P4´ as calculated. The amino acids are ordered from left to right: aromatic, aliphatic, hydrophilic, basic (positively charged) and acidic (negatively charged).

After the last biopanning, 120 individual phage clones were picked as individual plaques and the region encoding the randomly synthesized nona-peptides were PCR amplified. The 96 clearest PCR bands were selected for sequencing, and the nucleotide sequences were then translated into nona-peptides and aligned (Fig 2). Based on the alignments, the distribution of aa in positions P4 to P4´ were calculated (Fig 3). Of the 96 clones sequenced 83 provided good readable sequences. Alignment of these sequences resulted in a relatively well-defined extended specificity. All of these clones contained a Phe or a Tyr in the P1 position and a high number of aliphatic aa were seen both N and C terminal of this aromatic residue (Fig 2). Overall, very few acidic aa were found among the nonapeptide region of these 83 clones. A relatively broad selection of different aa was observed for the P1´position with high numbers of both hydrophilic aa as represented by Ser, Thr and Met, and also basic aa as primarily represented by Arg (Fig 2). In addition, aliphatic aa as Leu, Val, Ala and Gly were also seen at a relatively high number (Fig 2). This relatively broad spectrum of aa is also reflected in the frequency diagram of Fig 3. The relatively high number of His residues in positions P3´and P4´is somewhat artificial as after the random nonamer region comes a His-tag of six residues, which results in an overrepresentation of His residues in these two positions. The general pattern is a strict preference for Phe and Tyr in the P1 position, a high frequency of aliphatic aa both N and C terminal of the cleavage site from position P2 to P4 and P2´to P4´. In the P1’ position there was a relatively broad selection of aa. However, no aromatic and no negatively charged residues were observed in this position. In general we also observed a very low number of negatively charged aa in the entire region from P4 to P4´. The phage display results from the analysis of two additional rodent mucosal mast cell proteases rMCP-4 and mMCP-1 and the major connective tissue mast cell chymase in mouse mMCP-4 are also added to the figure for a comparison of similarities and differences in sequence preferences (Fig 2).

### Verifying the consensus sequence by the use of recombinant protein substrates

**Fig 4:**
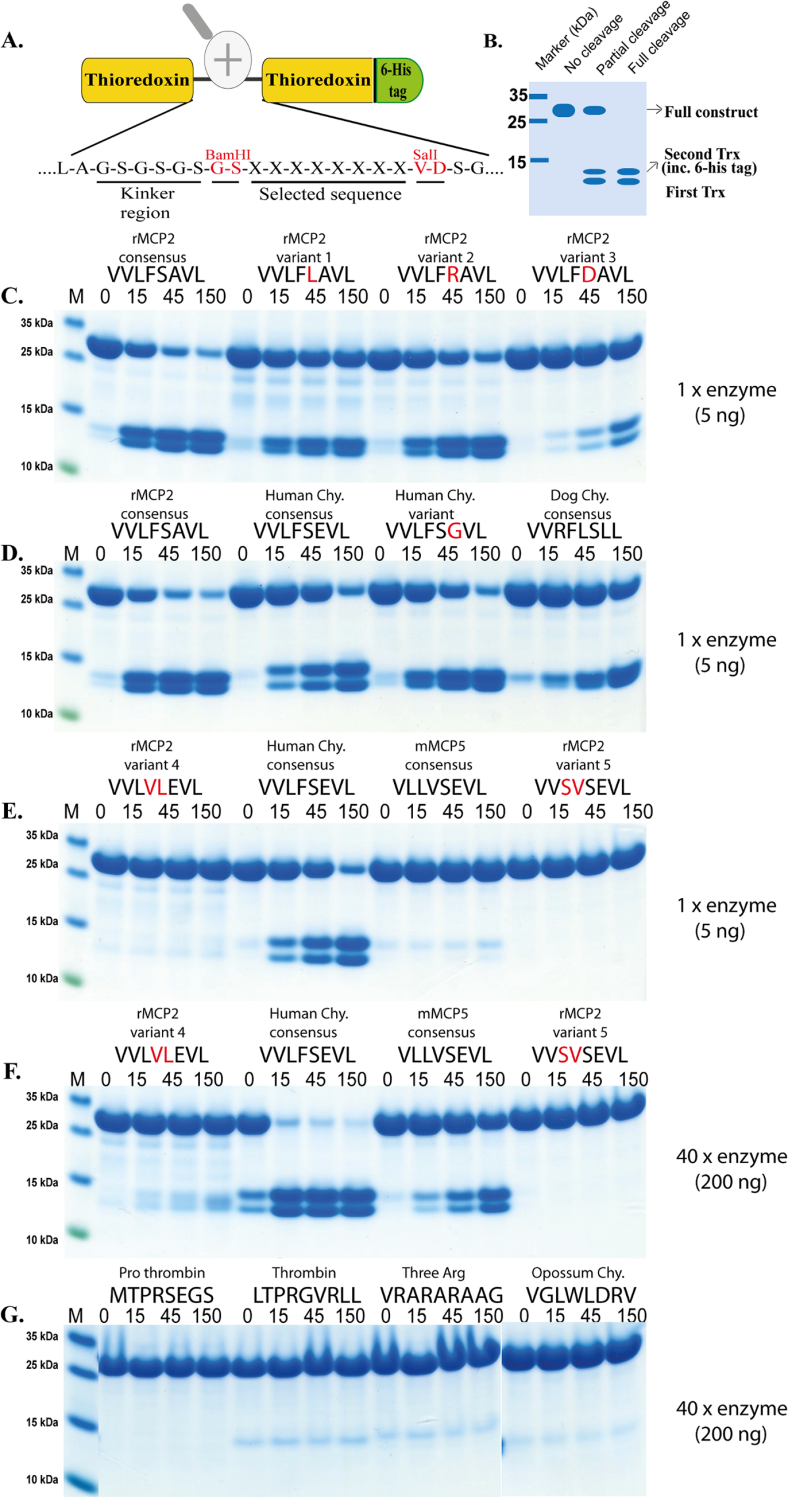
Analysis of the cleavage specificity by the use of recombinant protein substrates. Panel A shows the overall structure of the recombinant protein substrates used for analysis of the efficiency in cleavage by the MC. In these substrates two thioredoxin molecules are positioned in tandem and the proteins have a His_**6**_-tag positioned in their C termini. The different cleavable sequences are inserted in the linker region between the two thioredoxin molecules by the use of two unique restriction sites, one Bam HI and one SalI site, which are indicated in the bottom of panel A. Panels C-G show the cleavage of a number of substrates by rMCP-2. The name and sequence of the different substrates are indicated above the pictures of the gels. The time of cleavage in minutes is also indicated above the corresponding lanes of the different gels. The uncleaved substrates have a molecular weight of approximately 25 kDa and the cleaved substrates appear as two closely located bands with a size of 12–13 kDa (Panel B).

In order to verify the results from the phage display analysis and to get quantitative estimates of the importance of particular amino acids in positions surrounding the cleavage site we used a new type of recombinant substrate. The consensus sequence obtained from the phage display analysis was first inserted in a linker region between two *E*.*coli* thioredoxin molecules by ligating a double stranded oligonucleotide encoding the actual sequence into a BamHI and a SalI site of the vector construct (Fig 4A). For purification purposes a His_6_-tag was added to the C-terminal of this protein (Fig 4A). A number of related and unrelated substrate sequences were also later produced with this system, by ligating the corresponding oligonuclotides into the BamHI/SalI sites of the vector. All of these substrates were expressed as soluble proteins in a bacterial host, *E*.*coli* Rosetta gami, and purified on IMAC columns to obtain a protein with a purity of 90–95%. These recombinant proteins were then used to study the preference of rMCP-2 for these different sequences (Fig 4B–4G). The results showed that rMCP-2 very efficiently cleaved the consensus sequence obtained from the phage display analysis (VVLFSAVL). By changing the serine residue in the P1´position into a leucine (VVLF**L**GVL) the efficiency in cleavage by rMCP-2 drops significantly by a factor of 10 (Fig 4C). In contrast, change in the same position, P1´, to an arginine (VVLF**R**GVL) only marginally affected the efficiency of cleavage, by a factor 2–3 (Fig 4C). In contrast, changing the same position into a negatively charged aa, aspartic acid (VVLF**D**GVL), had a major effect on cleavage (Fig 4C). The activity dropped by approximately 20 times. This showed that negatively charged aa are not liked in the near vicinity of the cleavage site. Testing a few additional substrates that previously have been identified as optimal substrates for other MC enzymes, the human chymase (VVLFSEVL), the human chymase double mutant (human chymase variant VVLFS**G**VL) and the dog chymase (VVRFLSLL), showed that all three were cleaved relatively efficiently [26–28]. They were cleaved at a rate of three to ten times less efficiently compared to the rMCP-2 consensus (Fig 4D). The dog chymase sequence was the least effectively cleaved with approximately 10 times lower cleavage rate (Fig 4D). Changing the aromatic aa at the P1 position, the cleavage site, to a valine, dramatically lowered the cleavage activity. Almost no cleavage was seen with three substrates having a valine in the cleavable position (VVLVLEVL, VLLVSEVL and VVSVSEVL) (Fig 4E). However, when increasing the enzyme concentration by 40 times we observed cleavage of one of them (VLLVSEVL) possibly by low efficiency cleavage at double leucines. Testing 4 additional substrates, the activation site in prothrombin (MTPRSEGS), the human thrombin consensus cleavage site (LTPRGVRL), a site with three arginines (VRARARAAG) and the opossum MC chymase consensus cleavage site (VGLWLDRV) that has a tryptophan in the P1 position, did not result in any detectable cleavage even when using the higher enzyme concentration (40x) (Fig 4G) [29, 30]. This showed the very high specificity of the enzyme. Two aromatic aa, phenylalanine and tyrosine were strongly favored at the P1 position and another aromatic aa acid tryptophan, or valine or any other aa with the possible exception of multiple leucines were not tolerated. Negatively charges in or around the cleavage sites also had a very negative effect on the cleavage activity.

### Using the consensus cleavage site and related sequences to screen the rat proteome for potential in vivo substrates

A relatively good view of the preference for this enzyme had been obtained from the phage display analysis and the confirmation performed with the recombinant substrates. Using the consensus sequence obtained from these studies we designed a four amino acid long sequence to screen the entire rat proteome for potential *in vivo* substrates for rMCP-2. The 4 amino acid sequence had the following sequence: P2 (L/V), P1 (F/Y), P1' (S/A/V/R), P2' (A/S/V), which resulted in 48 different variants. Using this sequence for the screening of the rat proteome resulted in 4825 rat specific hits. To try to limit this number to enable a more detailed analysis, nuclear and cytoplasmic proteins were removed, as they do not represent likely *in vivo* targets. Among these originally 4825 hits, 78 were considered to be particularly interesting and could be considered as potential substrates for rMCP-2 (Table 1). As can be seen from this table a large fraction of these potential targets were cell adhesion or cell junction proteins and thereby to be potential targets in increasing permeability of the rat intestinal epithelium observed from several previous studies [15, 31]. Of interest were also the IL-9 and the angiotensin II receptors as they may represent regulatory feedback mechanisms to limit excessive expansion of MMCs by IL-9 and blood pressure control by angiotensin II.

**Table 1:** Identification of potential in vivo targets for rMCP-2.

Protein	NCBI accession No.	Motif	P1 position
lymphocyte antigen 6 complex locus protein G6d precursor	NP_001001970	GIL**F**SALL	10
Transmembrane protein 196	NP_001037734	MIL**F**SACC	72
Transmembrane protein 18	NP_001007749	SLV**F**SAPL	97
PREDICTED: CD177 antigen-like isoform X2	XP_008772673	VQV**F**SAGP	72
tetraspanin-18	NP_001101220	TDV**F**SATW	134
junctional adhesion molecule C precursor	NP_001004269	TLV**F**SAVH	206
integrin beta 4, isoform CRA_b	EDM06633	RLV**F**SALG	261
cell adhesion-like molecule AAA40858	AAA40858	WIV**F**SATT	10
PREDICTED: junctional adhesion molecule C isoform X1	XP_008764276	TLV**F**SAVH	241
transmembrane protein 10, isoform CRA_a	EDL94196	HFL**Y**SARL	40
claudin-3	NP_113888	KIL**Y**SAPR	197
cadherin-related neuronal receptor 1	BAB61763	LLL**Y**SALR	218
PREDICTED: transmembrane protein C1orf162 homolog	XP_006224300	KCL**F**SSSG	20
transmembrane protein 170B	NP_001008774	WAL**F**SSLF	44
PREDICTED: synapsin-3-like	XP_008763510	SSL**F**SSFS	60
PREDICTED: immunoreceptor Ly49i3 isoform X1	XP_008761658	NPL**F**SSDG	30
PREDICTED: membrane-spanning 4-domains subfamily A member 5	XP_003753436	ISL**F**SSIL	183
protocadherin alpha 10	AAT77554	PPL**F**SVAE	163
PREDICTED: leukocyte immunoglobulin-like receptor subfamily A member 5 isoform X1	XP_008772646	QAL**F**SVGP	78
V-set and transmembrane domain-containing protein 5 precursor	NP_001138342	IQL**F**SVGV	107
transmembrane and ubiquitin-like domain-containing protein 1	NP_001073622	TVL**F**SVLA	15
PREDICTED: transmembrane protein PVRIG isoform X1	XP_008757545	LTL**F**SVSR	160
cd86 antigen, isoform CRA_b	EDM11276	GIL**F**SVLA	7
		TKL**F**SVSI	189
desmocollin-3	NP_001100872	PGL**F**SVH	299
		ALL**F**SVLL	709
protocadherin beta 6 precursor	NP_001014780	PGL**F**SVW	614
protocadherin beta 4	NP_001108074	PGL**F**SVW	614
protocadherin beta-3	NP_001014783	PGL**F**SVW	615
PREDICTED: protocadherin beta-14	NP_001102865	PGL**F**SVW	615
transmembrane protein 47	NP_001102787	VML**F**AAVV	120
cadherin-10	EDL82597	VVL**F**AALK	612
claudin-23	NP_001028234	VVL**F**AAGL	122
similar to FCRL, isoform CRA_c	EDM09238	QEL**F**AAPV	150
glycosylation dependent cell adhesion molecule 1, isoform CRA_b	EDL86774	VLL**F**ASLA	9
Fcgr3-related sequence	AAU06142	CLL**F**AVDT	25
transmembrane protein 107	NP_001103118	LGL**F**AVEL	64
PREDICTED: killer cell lectin-like receptor 7	XP_008774079	LRL**F**AVAM	59
PREDICTED: high affinity immunoglobulin epsilon receptor subunit alpha isoform X1	XP_008768002	VIL**F**AVDT	159
syndecan-4	AFC78697	GIL**F**AVFL	157
epithelial membrane protein 3	NP_110474	VLL**F**VATL	20
protocadherin alpha 9 homolog, partial	AAT77565	KNL**F**VSET	12
epithelial membrane protein 1	NP_036975	IML**F**VSTI	20
killer cell lectin-like receptor, family E, member 1	NP_852037	SVL**F**VVCL	75
epithelial membrane protein 1	NP_036975	AGL**F**VVHI	9
small integral membrane protein 8 isoform 3	NP_001188305	TTL**F**RAVN	34
integrin alpha-7 precursor	NP_110469	AAL**F**RAR	469
protocadherin alpha 4	AAT77560	PRL**F**RVA	64
PREDICTED: collagen alpha-1(VI) chain	XP_001079629	RHL**F**RVP	1010
laminin chain, partial	CAA70095	QDV**F**SSAR	57
PREDICTED: CD177 antigen-like	XP_006228571	AQV**F**SSGP	64
CD209a molecule	NP_001099374	LTV**F**SVLL	64
gap junction beta-3 protein	NP_062113	VFV**F**RVLV	31
hyaluronic acid binding protein 2	EDL94490	MLV**F**RVLL	8
type-2 angiotensin II receptor	NP_036626	RSV**F**RVPI	333
interleukin 9 receptor, partial	AAN76721	HPL**Y**SVYH	8
putative gap junction protein connexin 43.4,partial	AAZ38711	FHL**Y**AACK	47
gap juncion epsilon-1 protein	NP_001184021	FHL**Y**AACK	94
angiotensin II receptor, partial	AAA40738	FNL**Y**ASVF	113
angiotensin II receptor subtype AT1C, partial	AAB26936	FNL**Y**ASVF	80
mucin, partial	AAB53196	LEL**Y**ASLC	109
leukocyte immunoglobulin-like receptor, subfamily B, member 4 precursor	NP_001013916	KYL**Y**ASVK	270
angiotensin receptor AT1	AAB25505	FNL**Y**ASVF	109
sorting nexin-20	NP_001020170	RLL**Y**AVRA	166
sorting nexin 16	AAG25677	ASL**Y**VSRA	284
transmembrane protein with EGF-like and two follistatin-like domains 1	EDL78188	SIL**Y**VVPS	47
annexin A10	NP_001102580	YKL**Y**RAIH	257
similar to transmembrane protein induced by tumor necrosis factor alpha, isoform CRA_a	EDM13357	HRL**Y**RVKL	33
PREDICTED: junctional adhesion molecule-like isoform X2	XP_008774920	KHV**Y**SSIT	292
PREDICTED: paired immunoglobulin-like type 2 receptor alpha isoform X1	XP_008759057	NIV**Y**ASIS	66
PREDICTED: paired immunoglobulin-like type 2 receptor alpha	XP_001061953	NIV**Y**ASIS	256
sorting nexin-4	NP_001121022	MDV**Y**ASSI	286
gap junction protein, alpha 12, 47kDa (predicted)	EDM04584	MRV**Y**VAQL	202
gap junction channel protein connexin47	AAP04733	MRV**Y**VAQL	187
gap junction gamma-2 protein	NP_001094254	MRV**Y**VAQL	212
mucin 13, epithelial transmembrane	EDM11355	KTV**Y**VSVV	65
RecName: Full = Mucin-13; Short = MUC-13; Flags: Precursor	P97881	QTV**Y**VSVV	324
connexin 30.3o,partial	AAT78351	VLV**Y**VVAA	22
gap junction beta-4 protein	NP_446436	VLV**Y**VVAA	36
gap junction beta-3 protein	NP_062113	VLV**Y**VVAA	36

By screening of the entire rat proteome with a 4 amino acid peptide sequence having the following sequence: P2 (L/V), P1 (F/Y), P1' (S/A/V/R), P2' (A/S/V) resulted in 4825 rat specific hits. This sequence was derived from the rMCP-2 cleavage consensus sequence. Nuclear and cytoplasmic proteins were removed from the list, as they most likely do not represent likely *in vivo* targets. Among these originally 4825 hits, 78 were considered to be particularly interesting and could be considered as potential substrates for rMCP2 and these are listed in this table.

### Cleavage of rat intestinal epithelium by active recombinant rMCP-2 to identify potential targets

**Fig 5:**
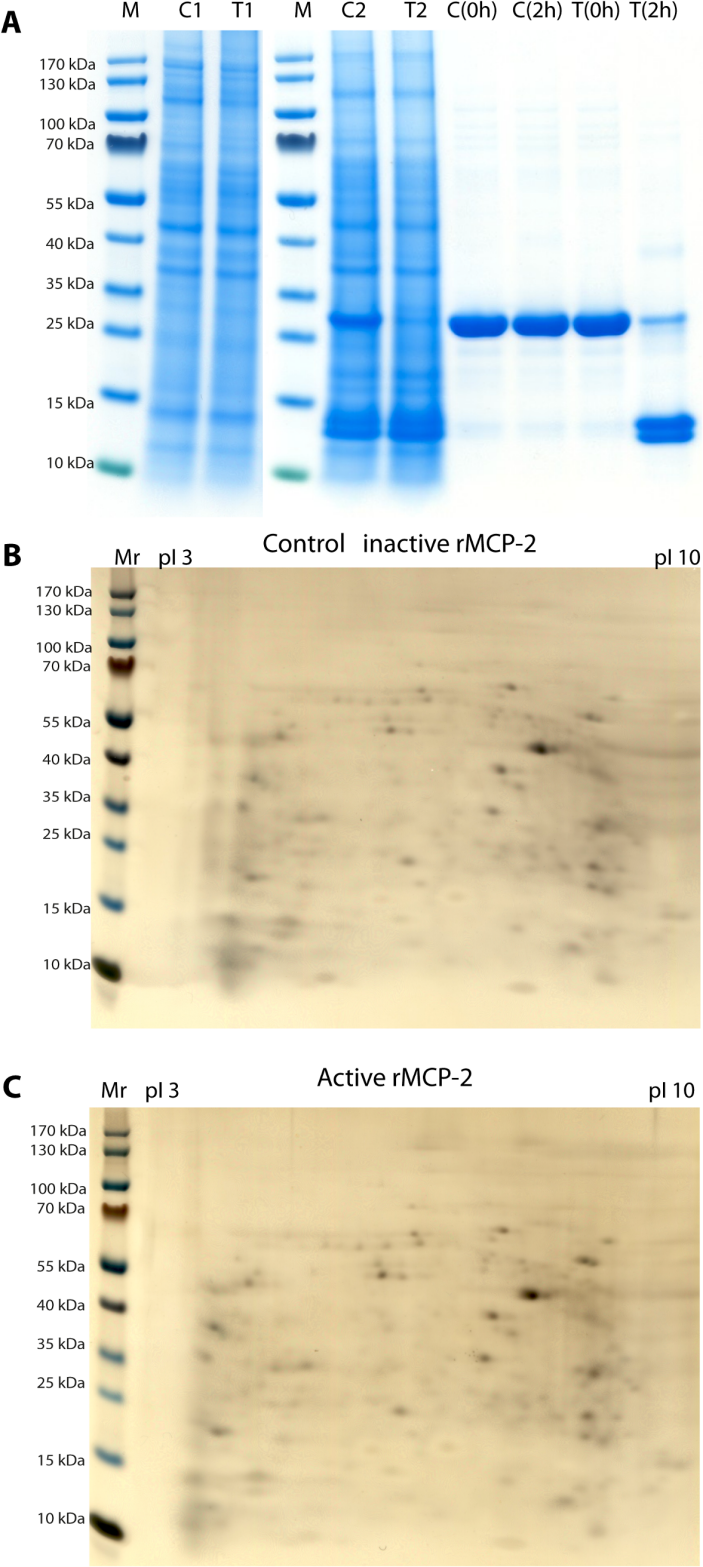
Analysis of the cleavage of purified rat intestinal epithelium by rMCP-2. Panel A shows a one dimensional gel of purified rat epithelial layer incubated for 2 hours with inactive (C1) and active enzyme (T1). In panels C2 and T2 have 10 ug of the recombinant substrate, the rMCP-2 consensus substrate (VVLFSAVL), been added to the cleavage reaction. The samples have then been incubated with inactive and active enzyme for 2 hours as for C1 and T1. In the left four lanes in panel A the recombinant substrate have been incubated with inactive enzyme (control) for 0 or 2 hours (C(0h) and C(2h)) and with active enzyme for 0 or 2 hours (T(0h) and T(2h)). As can be seen from the figure cleavage of the recombinant substrate can only be seen in the T(2h) lane. In panels B and C the results from the 2D gel analysis is shown. In panel B and C the inactive and active enzymes have been used, respectively.

As a next step in trying to identify potential intestinal targets we decided to test purified rat intestinal epithelium for its sensitivity to cleavage by rMCP-2. We used a recently published method to recover intact epithelial layers from rat intestines and thereby reduce the number of potential targets and the complexity of a 2D analysis [23]. Using a solution that dissolves the basement membrane combined with the air pressure makes it possible to isolate an intact epithelial layer as a sheet. This cell material was then exposed to active and inactive enzyme in separate reaction vials. The two samples were dissolved in sample buffer and separated on both one and two dimensional gels (Fig 5). By the 2D analysis an average of approximately 300 protein spots were reproducibly detected on gels by using silver staining (Fig 5). The proteins spanned a broad range of pI from 3 to 10 and apparent molecular mass from 10 to 100 kDa. However, after quantitative and qualitative analysis, no differential protein spots between rat intestinal epithelium incubated with inactive and active rMCP2 were detected, which have at least 2.0 fold increase or 0.5-fold decrease (Fig 5). As no difference could be detected the experiment was repeated and the consensus substrate was added to the tubes to ensure that the enzyme could cleave under these conditions (Fig 5A). The recombinant (thioredoxin) consensus substrate was efficiently cleaved under these conditions. However, we also observed some cleavage, although to a lesser degree, in the tube where inactive enzyme had been added indicating that active rMCP-2 or another enzyme with similar specificity was present in the tissue sample. One possibility would have been to add protease inhibitors to the sample during preparation. However, then the sample would have been swamped with inhibitors, which would most likely affected the active rMCP-2 when added to the sample. We therefore decided to try to look at pure recombinant cell adhesion and junction proteins for their susceptibility to cleavage by rMCP-2.

### Cleavage of a selection of recombinant cell adhesion and junction proteins by rMCP-2

As described above, the cleavage of intact rat intestinal epithelium was unsuccessful. We therefore decided to analyze a few selected cell adhesion and junction proteins expressed in the intestinal region for their sensitivity to cleavage by rMCP-2. Rat cadherin-1 (E-cadherin), rat cadherin-17 (VE-cadherin) and human protocadherin alpha-4 were three very interesting potential targets that were available as recombinant proteins produced in mammalian cells by commercial companies [31–33]. Based on a sequence comparison human and rat protocadherin alpha 4 was very similar in sequence, therefore the human sequence could also give a good estimate of the sensitivity of the rat protein to cleavage by rMCP-2.

**Fig 6:**
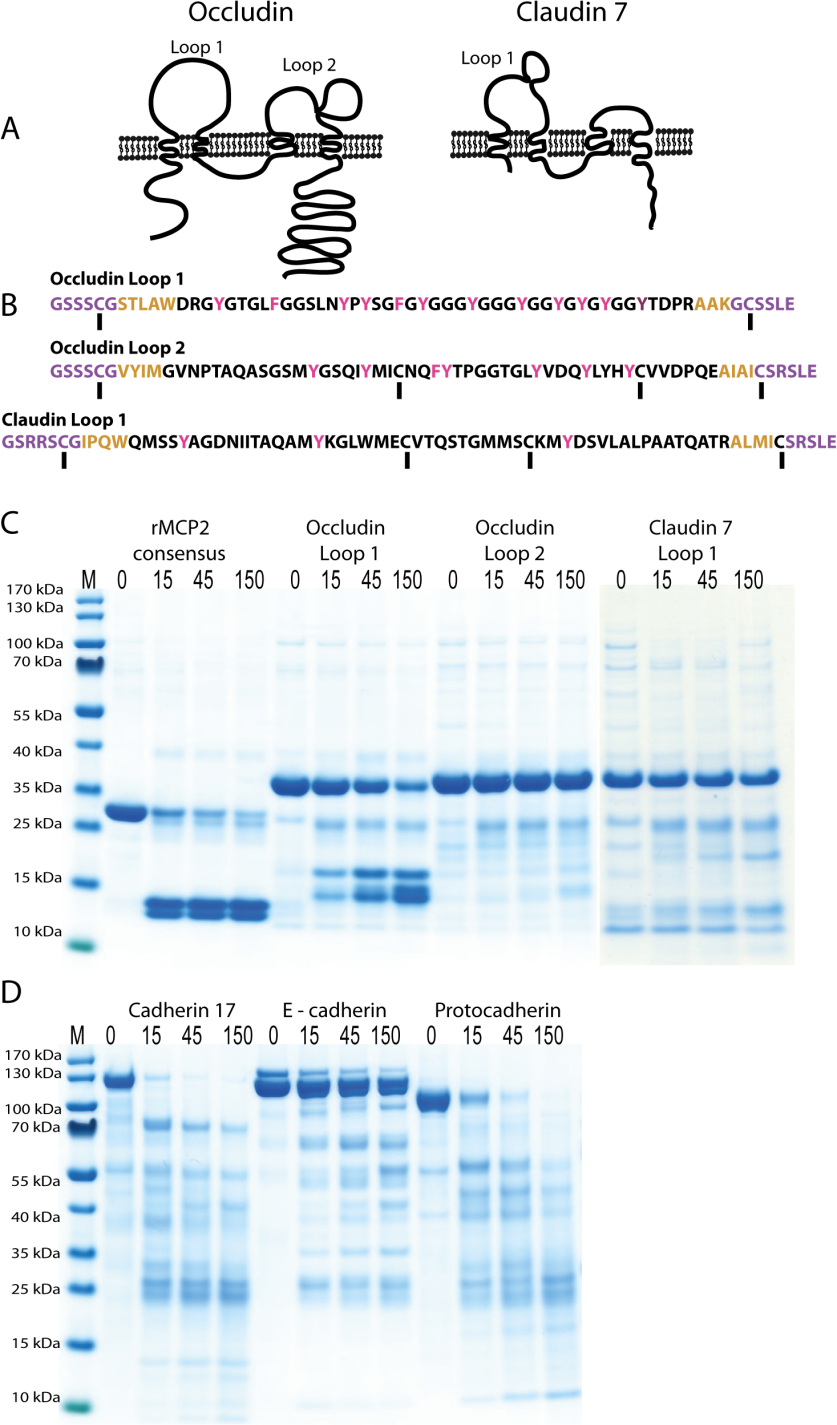
Analysis of the cleavage by active rMCP-2 of a number of recombinant cell adhesion and cell junctional proteins. Panel A shows schematic drawings of the basic structure of occludin and claudin. The sequence of the surface loops of occludin and claudin 7 are shown in panel B. The extracellular region is shown in black where the potential rMCP-2 cleavage sites (Tyr and Phe) have been marked in red. The yellow region shows the short region of the membrane spanning region remaining in these clones and the purple region is the kinker including a Cys that has been inserted to form a loop structure of the extracellular loops in the 2xTrx construct. In panel C the cleavage pattern of the rMCP-2 consensus 2xTrx substrate (VVLFSAVL), as positive control, occluding loop 1, occluding loop 2 and claudin 7 loop 1 is depicted. In panel D the cleavage of rat cadherin 17, rat E-cadherin and human protocadherin alpha 4 is presented where the protein is marked above each cleavage reaction and the time of cleavage in minutes is depicted above each lane. These three latter recombinant proteins were produced in mammalian cells. As can be seen from the figure cadherin 17 and protocadherin alpha 4 are very sensitive to cleavage by rMCP-2 whereas almost no cleavage is seen for E-cadherin.

A protein that previously has been identified as potential target by rMCP-2 is occludin, as it forms the barrier in the epithelial layer of the intestine [31, 32]. Claudins are also found in close connection to occludin junctions and were also represented in the potential target list in Table 1. Claudin 7 has been shown to be expressed at high levels in mouse and/or rat intestines [33]. Both of these are complicated membrane proteins that span the membrane 4 times and are therefore difficult to produce in a soluble and properly folded form (Fig 6A). We therefore decided to produce the extracellular loops that can be targets for an enzyme that act from outside of the cell. The extracellular region including a few amino acids from the outer part of the membrane-spanning region was inserted in the two thioredoxin vector. In order to stabilize the construct and obtain a folding similar to the bended loop structure of the native protein we inserted two cysteine residues, one in each end of the loop (Fig 6B). These cysteines will with high certainty form a stable disulphide bond after opening the bacterial cell where it is produced. It may even form in the cells as the *E*.*coli* Rosetta gami has a lower reducing potential than other *E*.*coli* strains and therefore cysteine bridges may form already within the cell. Occludin has two larger extracellular loops, which we produced separately (Fig 6A and 6B). Only the larger loop of claudin 7 was produced in this form as the small loop is very small and does not contain any sequence that could be a potential target for rMCP-2. As can be seen from Fig 6, a rapid cleavage of loop 1 of occludin, protocadherin alpha 4 and cadherin 17 was observed, but no cleavage of loop 2 of occludin, loop 1 of claudin 7, and E-cadherin could be detected. Interestingly, loop 1 of occludin contains almost a perfect repeat of chymase substrate sequences, whereas loop 2 of occluding and loop 1 of claudin only contains a few relatively poor potential site and both of them also contain a putative disulphide link that may influence accessibility of these sites (Fig 6A).

## Discussion

Granule proteases of hematopoietic cells have been studied relatively intensively for many years and many potential substrates have been identified. However, it has been very difficult to show that they also are important *in vivo* targets. Some of the hematopoietic serine proteases have a relatively broad specificity, for example the neutrophil proteases, whereas others are more restrictive. The rodent mucosal MC proteases clearly belong to the latter category. Three of them have so far been studied in detail, the major mucosal protease in mice mMCP-1, one of the minor rat mucosal proteases rMCP-4 and now the major MMC protease in rat rMCP-2 [18, 34]. Based on both phage display and recombinant substrate analyses we can conclude that rMCP-2 is a highly specific protease. No cleavage was seen with the substrates lacking a Phe or a Tyr except one that was found to cleave at a low rate when adding 40 times more enzyme, which had double leucines at and before the P1 position. However, the aromatic aa Trp was not tolerated in the P1 position, where no cleavage was seen even after adding 40 times more enzyme (Fig 3). Interestingly also no preference for negatively charged aa in the P2´position was observed which is a characteristic feature of many connective tissue MC chymases including the human chymase, mMCP-4, rMCP-5 and the opossum chymase [19, 20, 26, 30].

The role of rMCP-2 and mMCP-1 in increasing intestinal permeability and thereby participating in parasite clearance has previously been well documented. However, the exact targets have not yet been identified. Likely targets are cell adherence and cell junction proteins and one potential target that fits quite well with the previous experiments is occludin as it is found at high concentrations at the epithelial lining of the gut [31, 32]. Several claudins are also expressed in different parts of the body and take part in cell junction functions [33]. A detailed screening for claudins that are expressed in various organs of mice has shown that the most widely distributed and most highly expressed claudin in mouse intestines is claudin 7. Claudin 18 is also expressed there but more variably in occurrence and amount [33]. We therefore decided to look at claudin 7. Intravenous injection of rMCP-2 or mMCP-1 has previously been shown to have a strong effect on gut permeability indicating that the protease both cleaves targets in the endothelial lining of the blood vessels and at the tight junctions of the epithelial cells of the intestine. These experiments also indicate a high selectivity, as the MMC proteases seem to do this primarily in the intestinal region. Selectivity for the basolateral, as opposed to the apical side, was also seen when analysing monolayers of epithelial cells for the effect by rMCP-2 [31]. Occludin is one potential target and this protein has two extracellular loops (Fig 6). One of them has a sequence with repeating tyrosines, indicating that it may have evolved as a perfect chymase substrate. In contrast the other loop of occludin has only a few relatively poor chymase sites and this loop also contain a potential disulphide bridge that may affect accessibility of these sites (Fig 6A). As seen from Fig 6 and as expected from the sequence, loop 1 was efficiently cleaved, whereas loop 2 was not cleaved even after extended incubation with the active enzyme. The same situation was observed for the large loop of claudin 7. No good chymase sites are present in this loop, and it also contains a putative cysteine bridge which may explain the resistance to digestion (Fig 6A). Two potential endothelial targets were also identified cadherin 17 (VE-cadherin) and protocadherin alpha 4. Interestingly, protocadherin alpha 4 was also identified together with a number of cell adhesion molecules in the screening of potential targets for mMCP-1, the mouse counterpart of rMCP-2, which further strengthen its importance for the permeability increasing effect by these two enzymes [34]. However, E-cadherin was not cleaved under these conditions (Fig 6). The latter finding also shows the selectivity of the enzyme. Some targets are cleaved very efficiently whereas others are left relatively intact. The substrates we see efficient cleavage of are most likely not the only ones cleaved by rMCP-2. However, they represent a few likely *in vivo* targets for this interesting enzyme. Several gap junction proteins were also identified in the screening, gap junction alpha, beta, gamma and a, indicating that these also may be important targets (Table 1). Although important for ion transport and signaling between cells, these may have little effect on intestinal permeability for macromolecules and cells. Not only one protocadherin but a number of them was also seen in the list and two additional claudins indicating that other variants of the claudin family may be interesting targets (Table 1). More work will be needed to map the fine specificity of the cell adhesion and cell junction proteins that are the most important targets for this enzyme. However, here we have defined at least three potential targets in this family of proteins that may serve as important *in vivo* targets for this enzyme.

A few interesting potential targets were also identified among the non-adhesion and non-junction proteins (Table 1). Three different angiotensin receptors were found in the list: type-2 angiotensin II receptor, angiotensin II receptor partial, angiotensin II receptor subtype AT1C partial and angiotensin receptor AT1 which may indicate that rMCP-2 can be involved in feedback regulation of angiotensin activity and thereby in blood pressure regulation. This is highly interesting in regards to the potential role of the human chymase, mMCP-4 and rat vascular chymase in generation of angiotensin II [35]. Do the mucosal and the connective tissue chymases have opposing functions in angiotensin regulation?

Another potential target identified was the IL-9 receptor (Table 1). IL-9 is known to be very important regulator of MMC expansion during parasite infection. TGF-β is expressed constitutively in the intestinal region by both stroma and T cells, most likely partly to limit excessive inflammation by intestinal microbial TLR agonists [2]. IL-9 produced by T_H_9 cells during the early stage of intestinal parasite infection is therefore most likely the key cytokine in regulating the expansion of MMCs seen at approximately a week after parasite infection [36, 37]. IL-25 and possibly also IL-1 and IL-33 seem to be of major importance for the production of IL-9, and Fox P3 regulatory cells may have a dampening effect on the IL-9 production [38, 39]. Here, rMCP-2 may have a role in controlling this increase in MMC number by cleaving the IL-9 receptor and thereby act as a negative feedback regulator, an interesting idea that needs to be verified experimentally.

In conclusion, we present a detailed analysis of the extended cleavage specificity of the major rat MMC protease rMCP-2, involving both phage display analysis and a panel of recombinant substrates to give a quantitative estimate on the involvement of the aa at and around the cleavage site. We have shown that this is one of the most specific hematopoietic serine proteases analyzed thus far, with a strong preference for Tyr and Phe at the P1 position and with a preference for aliphatic aa in the P2, P3, P4, P2’ and P3´positions, and a relatively broad selection of aa tolerated in the P1´position including, Ser, Thr, Arg and Ala. We also showed that this enzyme efficiently cleaves three endothelial or epithelial adhesion or junction proteins: loop 1 of occludin, cadherin 17 (VE-cadherin) and protocadherin alpha 4, indicating that these three may be the prime targets for the observed rapid increase in intestinal permeability upon mucosal MC granule release as response to parasite infections.
